# Large Language Models in Lung Cancer: Systematic Review

**DOI:** 10.2196/74177

**Published:** 2025-09-30

**Authors:** Ruikang Zhong, Siyi Chen, Zexing Li, Tangke Gao, Yisha Su, Wenzheng Zhang, Dianna Liu, Lei Gao, Kaiwen Hu

**Affiliations:** 1 Graduate School, Beijing University of Chinese Medicine, Beijing, China; 2 Oncology Department, Dongfang Hospital, Beijing University of Chinese Medicine, No. 6, Fangxingyuan 1st District, Fengtai District, Beijing, China, 86 13911650713

**Keywords:** lung cancer, LC, large language modeling, LLM, artificial intelligence, full-cycle management, clinical practice, systematic review, diagnosis, treatment

## Abstract

**Background:**

In the era of data and intelligence, artificial intelligence has been widely applied in the medical field. As the most cutting-edge technology, the large language model (LLM) has gained popularity due to its extraordinary ability to handle complex tasks and interactive features.

**Objective:**

This study aimed to systematically review current applications of LLMs in lung cancer (LC) care and evaluate their potential across the full-cycle management spectrum.

**Methods:**

Following PRISMA (Preferred Reporting Items for Systematic Reviews and Meta-Analyses) guidelines, we conducted a comprehensive literature search across 6 databases up to January 1, 2025. Studies were included if they satisfied the following criteria: (1) journal articles, conference papers, and preprints; (2) studies that reported the content of LLMs in LC; (3) including original data and LC-related data presented separately; and (4) studies published in English. The exclusion criteria were as follows: (1) books and book chapters, letters, reviews, conference proceedings; (2) studies that did not report the content of LLMs in LC; and (3) no original data, and LC-related data that are not presented separately. Studies were screened independently by 2 authors (SC and ZL) and assessed for quality using Quality Assessment of Diagnostic Accuracy Studies-2, Prediction Model Risk of Bias Assessment Tool, and Risk Of Bias in Non-randomized Studies - of Interventions tools, selected based on study type. Key data items extracted included model type, application scenario, prompt method, input and output format, outcome measures, and safety considerations. Data analysis was conducted using descriptive statistics.

**Results:**

Out of 706 studies screened, 28 were included (published between 2023 and 2024). The ability of LLMs to automatically extract medical records, popularize general knowledge about LC, and assist clinical diagnosis and treatment has been demonstrated through the systematic review, emerging visual ability, and multimodal potential. Prompt engineering was a critical component, with varying degrees of sophistication from zero-shot to fine-tuned approaches. Quality assessments revealed overall acceptable methodological rigor but noted limitations in bias control and data security reporting.

**Conclusions:**

LLMs show considerable potential in improving LC diagnosis, communication, and decision-making. However, their responsible use requires attention to privacy, interpretability, and human oversight.

## Introduction

Lung cancer (LC) is one of the leading causes of cancer incidence and mortality worldwide [1,2]. Early detection and accurate treatment are essential to improving survival [3,4], and low-dose computed tomography (CT) screening has been shown to reduce mortality [5,6]. In recent years, integrated full-cycle management—covering prevention, screening, diagnosis, treatment, and supportive care—has been promoted to improve both survival and quality of life [7,8]. However, this approach requires complex workflows and large-scale data processing, placing heavy demands on medical resources and personnel.

Artificial intelligence, particularly large language models (LLMs), offers a potential solution. LLMs can process complex clinical data, support decision-making, and enable personalized communication between patients and health care providers [9-11]. At the same time, they face limitations such as bias [12] and hallucinations [13]. These issues highlight the need for a systematic evaluation of their role in clinical practice.

Numerous studies have been conducted on LLMs in the field of LC. Some scholars have carried out a systematic review on the potential of LLMs and natural language processing in LC diagnosis [14]. However, it was limited to diagnostic applications, relied on outdated evidence, and lacked a comprehensive scope. This study aims to address these gaps by systematically reviewing the latest applications of LLMs in LC. We summarize current use cases, model types, fine-tuning strategies, limitations, and future directions. Our goal is to help clinicians and researchers better understand how to integrate LLMs into LC management while recognizing their potential and constraints.

## Methods

### Overview

This study was conducted in accordance with the PRISMA (Preferred Reporting Items for Systematic Reviews and Meta-Analyses) guidelines [15]. The PRISMA checklist is presented in Checklist 1.

### Eligibility Criteria

We established clear inclusion and exclusion criteria based on the research objectives, as summarized in Table 1. No time restrictions were applied during the selection of studies.

**Table 1.** Inclusion and exclusion criteria.

Criterion	Inclusion	Exclusion
Types of studies	Journal articles, conference papers, and preprints	Books and book chapters, letters, reviews, and conference proceedings
Content	Content involves LLMs^a^ and LC^b^	Neither LLMs nor LC
Outcomes	Including original data, and LC-related data are presented separately	No original data, and LC-related data are not presented separately
Language	English	Non-English

a LLM: large language model.

b LC: lung cancer.

### Data Sources

Eligible studies were identified by searching 6 electronic databases: PubMed, Web of Science, IEEE, Embase, Cochrane Library, and Scopus. The final search was run up to January 1, 2025.

### Search Strategy

The search strategy was structured as follows: ((“large language model”) OR (“LLM”) OR (“ChatGPT”) OR (“chatGPT”)) AND ((“lung cancer”) OR (“lung tumor”) OR (“pulmonary ground-glass”) OR (“lung malignancy”) OR (“lung carcinoma”) OR (“lung metastasis”) OR (“lung metastatic”) OR (“pulmonary metastatic”) OR (“pulmonary metastasis”)).

### Selection Process

EndNote X9.3.3 (build 13966; Clarivate) was used to manage references and remove duplicates. Two authors (RZ and SC) independently screened the titles and abstracts, followed by full-text screening based on the predefined inclusion and exclusion criteria. Discrepancies were resolved through discussion, with arbitration by a third author (ZL) when necessary. The consistency degree of the 2 authors was verified using the kappa consistency test.

### Data Collection Process

Two authors (RZ and SC) carried out the data collection process. All extracted data from the main text, tables, figures, and appendices were annotated using WPS Office Excel (version 12.1.0.18608; Kingsoft Office Software).

### Data Items

The data extraction form included the following items: title, first author, year of publication, study design, LLM model used, application scenario, intervention, prompt engineering approach, input and output formats, and outcome measures. The consistency rate of the 2 authors was calculated.

### Quality Appraisal

To ensure a rigorous evaluation of study quality, we adopted a mixed methods approach based on the framework by Omar and Levkovich [16]. Appropriate quality assessment tools were selected based on the specific application of LLMs in each study. QUADAS-2 (Quality Assessment of Diagnostic Accuracy Studies-2) [17] is a validated and widely accepted tool for evaluating the quality of diagnostic tests. For studies where LLMs were primarily applied to LC diagnosis or staging, the QUADAS-2 tool was used. PROBAST (Prediction Model Risk of Bias Assessment Tool) [18] is specifically designed to assess the risk of bias in studies involving predictive modeling and was applied accordingly. The ROBINS-I (Risk Of Bias in Non-randomized Studies - of Interventions) [19] tool is commonly used to assess bias in observational studies. For research on information extraction and knowledge-based tasks, these were considered observational in nature, and thus, the ROBINS-I tool was applied. Given that studies involving LLMs differ in format and content from conventional clinical trials, 2 oncology experts at the chief physician level (LG and KH) adapted the criteria of each tool accordingly to better reflect the nature and objectives of the included studies.

The quality assessment was carried out back-to-back by 2 researchers (SC and ZL) and, in the case of controversial content, by a third researcher (RZ) in order to deliberate jointly on the decision. The final results are reviewed by 2 experts (LG and KH). The consistency degree of the 2 authors was verified using the kappa consistency test.

### Synthesis Methods

Meta-analysis was not planned in this review. We conducted data analysis using descriptive statistics. Frequencies were used to summarize the application scenarios, prompt strategies, and other relevant characteristics of LLMs. Narrative synthesis was conducted due to the heterogeneity in the specified aims and methodologies across the included studies. We primarily used WPS and the BioRender website for figure generation. We used IBM SPSS (version 29.0.2.0) to calculate the kappa value.

## Results

### Search Results

**Figure 1.**
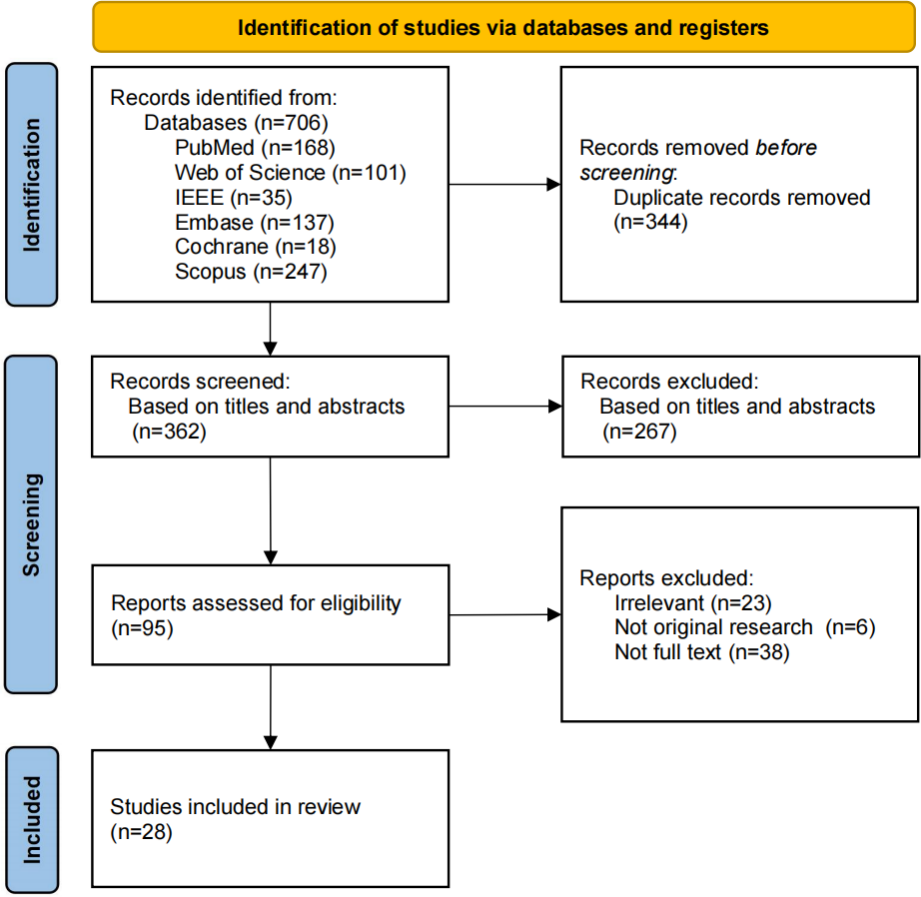
Study flowchart (produced according to the PRISMA [Preferred Reporting Items for Systematic Reviews and Meta-Analyses] 2020 flow diagram).

In this study, a total of 706 studies were retrieved, and 28 studies [20-47] were finally included after screening. The kappa values of the 2 researchers during the screening stage were 0.87, indicating good consistency. The specific screening process is presented in Figure 1.

### Basic Information of Included Sources

During the data extraction stage, the consistency rate of the 2 authors reached 0.97. All included studies were published between 2023 and 2024, with 7 published in 2023 [21,23,26,29,31,32,40] and 21 in 2024 [20,22,24,25,27,28,30,33-39,41-47]. Of these, 13 studies originated from the United States [20,23-27,29,30,32,36,43,45,46], followed by 3 each from South Korea [33,42,44], Germany [21,31,34], and China [22,35,39]. The remaining studies were conducted in India [38,47], Turkey [28], Japan [37], Greece [40], and the Netherlands [41]. Publication types included 5 conference papers [23,35,38,40,47] and 4 preprints [24,25,27,39]. The most commonly used LC type was non–small cell lung cancer (NSCLC). Most studies focused on knowledge-based question answering, information extraction, and diagnostic support. The LLMs used varied widely, with frequent use of OpenAI’s GPT-3.5, GPT-4, and GPT-4V, Meta AI’s LLaMA-2, and Google AI’s Bard. A summary of these details is provided in Table 2.

**Table 2.** Summary of included sources.

Study	Title	Country	Device	Best performance
Information extraction				
Bhattarai et al [20]	Leveraging GPT-4 for identifying cancer phenotypes in electronic health records: a performance comparison between GPT-4, GPT-3.5-turbo, Flan-T5, Llama-3-8B, and spaCy’s rule-based and machine learning–based methods	United States	GPT-4, GPT-3.5-turbo, Flan-T5, Llama-3-8B, spaCy	GPT-4
Fink et al [21]	Potential of ChatGPT and GPT-4 for data mining of free-text CT^a^ reports on lung cancer	Germany	ChatGPT, GPT-4	GPT-4
Hu et al [22]	Zero-shot information extraction from radiological reports using ChatGPT	China	ChatGPT	—^b^
Naik et al [23]	Applying large language models for causal structure learning in non–small cell lung cancer	United States	NR^c^	—
Niu et al [24]	Cross-institutional structured radiology reporting for lung cancer screening using a dynamic template-constrained large language model	United States	Llama-3.1 (8B, 70B, 405B), Qwen-2 (72B), Mistral-Large (123B)	Llama-3.1 (8B, 70B, 405B)
Lee et al [25]	SEETrials: leveraging large language models for safety and efficacy extraction in oncology clinical trials	United States	GPT-4	—
Lyu et al [26]	Translating radiology reports into plain language using ChatGPT and GPT-4 with prompt learning: results, limitations, and potential	United States	ChatGPT	—
Knowledge-based question and answer evaluation				
Ferrari-Light et al [27]	Evaluating ChatGPT as a patient resource for frequently asked questions about lung cancer surgery–a pilot study	United States	GPT-3.5	—
Gencer [28]	Readability analysis of ChatGPT’s responses on lung cancer	Turkey	GPT-3.5-turbo	—
Haver et al [29]	Use of ChatGPT, GPT-4, and Bard to improve readability of ChatGPT’s answers to common questions about lung cancer and lung cancer screening	United States	ChatGPT, GPT 4, Bard	Bard
Janopaul-Naylor et al [30]	Physician assessment of ChatGPT and Bing answers to American Cancer Society’s questions to ask about your cancer	United States	GPT-3.5, Bing AI	GPT-3.5
Rogasch et al [31]	ChatGPT: can you prepare my patients for [18F]FDG PET/CT and explain my reports?	Germany	ChatGPT	—
Rahsepar et al [32]	How AI responds to common lung cancer questions: ChatGPT versus Google Bard	United States	GPT-3.5, Google Bard experimental version	GPT-3.5
Auxiliary diagnosis				
Cho et al [33]	Extracting lung cancer staging descriptors from pathology reports: a generative language model approach	Korea	Llama-2-7B, Mistral-7B, Deductive Llama-2-7B (Orca-2), Deductive Mistral-7B (Dolphin), AWS Llama-2-70B, AWS Titan express	Deductive Mistral-7B
Dehdab et al [34]	Evaluating ChatGPT-4V in chest CT diagnostics: a critical image interpretation assessment	Germany	GPT-4V	—
Hu et al [35]	The power of combining data and knowledge: GPT-4o is an effective interpreter of machine learning models in predicting lymph node metastasis of lung cancer	China	GPT-4	—
Huang et al [36]	A critical assessment of using ChatGPT for extracting structured data from clinical notes	United States	GPT-3.5-Turbo-16k	—
Yasaka et al [37]	Fine-tuned large language model for extracting patients on pretreatment for lung cancer from a picture archiving and communication system based on radiological reports	Japan	Transformers Japanese model	—
Vallabhaneni et al [38]	Improved lung cancer detection through use of large language systems with graphical attributes	India	NR	—
Qu et al [39]	The rise of AI language pathologists: exploring two-level prompt learning for few-shot weakly-supervised whole slide image classification	China	GPT-4	—
Panagoulias et al [40]	Evaluation of ChatGPT-supported diagnosis, staging and treatment planning for the case of lung cancer	Greece	ChatGPT	—
Mithun et al [41]	Transfer learning with BERT and ClinicalBERT models for multiclass classification of radiology imaging reports	Netherlands	BERT, ClinicalBERT	ClinicalBERT
Lee et al [42]	Lung cancer staging using chest CT and FDG PET/CT free-text reports: comparison among three ChatGPT large-language models and six human readers of varying experience	Korea	GPT-4o, GPT-4, GPT-3.5	GPT-4o
Treatment decision-making				
Dong et al [43]	Large-language-model empowered 3D dose prediction for intensity-modulated radiotherapy	United States	Llama-2	—
Jeong et al [44]	The prediction of stress in radiation therapy: integrating artificial intelligence with biological signals	Korea	Decision tree, random forest, support vector machine, LSTM^d^, GPT-4, GPT-3.5	LSTM (limited information); GPT-4 (complex and diverse information)
Aided nursing				
Dos Santos et al [45]	An example of leveraging AI for documentation: ChatGPT-generated nursing care plan for an older adult with lung cancer	United States	ChatGPT	—
Scientific research				
Wang et al [46]	Scientific figures interpreted by ChatGPT: strengths in plot recognition and limits in color perception	United States	GPT-4V	—
Devi et al [47]	Automating clinical trial eligibility screening: quantitative analysis of GPT models versus human expertise	India	GPT-3.5-turbo	—

a CT: computed tomography.

b Not available.

c NR: not reported.

d LSTM: long short-term memory.

Notably, many studies used multiple LLMs or conducted comparative evaluations, and some explored multimodal capabilities such as image interpretation. The best-performing models identified in these comparative studies are summarized in Table 2. The results indicate that the ChatGPT (OpenAI) series models are the most comprehensive and widely applicable, exhibiting strong performance in both information extraction and auxiliary diagnosis, highlighting the improvements achieved through version updates. However, for a limited number of tasks or under constrained information conditions, lightweight models, such as Bard or architectures like long short-term memory networks may perform better. In addition, LLMs specialized in the medical domain, such as Deductive Mistral-7B and ClinicalBERT, demonstrate superior performance compared with general-purpose pretrained models.

### Prompt Engineering and Model Training

Prompt engineering plays a critical role in the development and application of LLMs and is a frequent topic of discussion in related studies. Therefore, we synthesized and summarized the prompt engineering strategies, model inputs and outputs, and evaluation metrics used in the included studies (Table 3). In total, 12 (43%) studies [24,27-30,32,34,37,38,41,44,47] did not explicitly describe their prompting strategies, which were generally basic queries, primarily intended for educational use. Furthermore, 16 (57%) studies [20-23,25,26,31,33,35,36,39,40,42,43,45,46] clearly described their prompting methods. These methods included prompt templates, instructional prompts, zero-shot or few-shot learning, and other fine-tuning techniques. Regarding the types of training data, a total of 22 (79%) studies [20-23,25,26,31,33,35,36,39,40,42,43,45,46] focused on text, 3 (11%) studies [24,34,43] on images, and 3 (11%) studies [38,42,46] on a combination of images and text. Outcome metrics commonly included confusion matrices, rating scales, and comparisons against gold-standard references or expert consensus. Some studies also reported on the time efficiency and cost-effectiveness of LLM-generated outputs.

**Table 3.** Prompt engineering and model training.

Study	Prompt method or content	Model input	Model output	Outcome indicators
Information extraction				
Bhattarai et al [20]	Zero-shot prompt	Segmented text and zero-shot prompt	Phenotypic information (cancer staging, cancer treatment), evidence of cancer recurrence, and organs affected by cancer recurrence	Accuracy, recall rate, *F*_1_-score, generation time, operating costs
Fink et al [21]	25 original lung cancer CT^a^ reports used to prompt training	Original lung cancer CT reports	Tumor information includes tumor lesions, metastatic sites, tumor impression assessment (deterioration, stability, improvement), and interpretation	McNemar test, accuracy, 5-point Likert scale
Hu et al [22]	Prompt template, including an information extraction command, a question form, extraction requirements, and some relevant medical knowledge	CT reports and prompt template	Answers to the question form	Accuracy, precision, recall rate, and *F*_1_-score
Naik et al [23]	Code interpreter plugin (developed by OpenAI)	Electronic medical records, genomic data	Directed acyclic graph	Bdeu score
Niu et al [24]	Not mentioned	CT imaging	Standardized and structured radiological reports	*F*_1_-score, CI, McNemar test, and *z* test
Lee et al [25]	Prompt templates	Journal abstract	Details of clinical trials in the article	Accuracy, recall rate, *F*_1_-score
Lyu et al [26]	Instruction	Radiological reports	Report translation and suggestions	Self score, report completeness and accuracy
Knowledge-based question and answer evaluation				
Ferrari-Light et al [27]	Not mentioned	Questions	Answers	5-point Likert scale
Gencer [28]	Not mentioned	Questions	Answers	Flesch Reading Ease (FRE) formula, Flesch-Kincaid Grade level (FKGL), Gunning FOG formula, SMOG index, Automated readability index (ARI), Coleman-Liau index, Linsear write formula, Dale-Chall readability score, Spache readability formula
Haver et al [29]	Not mentioned	Questions	Baseline responses and simplified responses	Reading Ease Score, readability, clinical appropriateness
Janopaul-Naylor et al [30]	Not mentioned	Questions	Answers	Self rating
Rogasch et al [31]	Regeneration-response function repeated three times for training	Questions	Answers	Self rating
Rahsepar et al [32]	Not mentioned	Questions	Answers	Accuracy, consistency
Auxiliary diagnosis				
Cho et al [33]	Morphology group	Segmented pathological report	42 lung cancer staging descriptors; tumor node classification	Macro *F*_1_-score, accurate matching ratio, accuracy
Dehdab et al [34]	Not mentioned	CT images of lung window	Diagnosis of lung cancer (yes or no)	Accuracy, sensitivity, specificity
Hu et al [35]	Prompt templates, including roles, tasks, patient data, machine learning model results and instructions	Prompt templates	Prediction results of lymph node metastasis in lung cancer	AUC^b^, AP (average precision of 3 repetitions)
Huang et al [36]	Prompt templates, including clinical staging introduction and instructions	Pathology reports and prompt templates	Tumor size, tumor characteristics, lymph node involvement, histological classification, clinical staging	Accuracy, average precision, *F*_1_-score, Kappa, recall rate
Yasaka et al [37]	Not mentioned	Clinical indications and diagnosis of radiological reports	Patient grouping (Group 0: no lung cancer, Group 1: lung cancer pre-treatment present, Group 2: after lung cancer treatment, Group 3: planned radiotherapy)	Overall accuracy, sensitivity, consistency, AUC, classification time
Vallabhaneni et al [38]	Not mentioned	Images, symptoms, clinical prescriptions	Diagnosis of lung cancer (yes or no)	Accuracy, recall rate, *F*_1_-score, AUC
Qu et al [39]	Guide GPT-4 to visually describe complex medical concepts	Questions (text)	Answers	AUC
Panagoulias et al [40]	Build and refine prompts based on the returned answers	Symptom description	Diagnosis and treatment plan for lung cancer	Self-drafted standards
Mithun et al [41]	Not mentioned	Radiological reports	Classification results of lung cancer	AUC, *F*_1_-score, accuracy, recall rate, precision
Lee et al [42]	Instruction	Chest CT and FDG PET^c^ or CT reports	The maximum size of the primary tumor, local invasion, satellite lesions, metastatic lymph nodes, intrathoracic and extrathoracic metastases, and TNM^d^ staging diagnosis	Accuracy, recall rate, *F*_1_-score, average task completion time, misreading rate
Treatment decision-making				
Dong et al [43]	Clinical physician commands (findings, treatment goals, and precautions)	CT images	DVH (Radiation dose volume histogram)	Mean absolute error (MAE) of Dmax, Dmean, D95, and D1 between actual and predicted plans
Jeong et al [44]	Not mentioned	Biological signals before radiotherapy and instructions	Prediction results of biological signals and stress response during radiotherapy	Accuracy, recall rate, precision, *F*_1_-score
Aided nursing				
Dos Santos et al [45]	Patient’s needs framework (Situation or Background, Physical, Safety, Psychosocial, Spiritual or Culture, Nursing Recommendation)	Medical records, needs framework, problem prompts	Care plan	The number of items that match the gold standard (16 tags including NANDA, NOC, and NIC)
Scientific research				
Wang et al [46]	Instruction	K-M^e^ curves generated based on gene expression data and survival information	Analysis and Interpretation of K-M curves	Overall accuracy, Accuracy under each category
Devi et al [47]	Not mentioned	Unprocessed raw dataset	Whether the patient is qualified for enrollment (yes or no)	Accuracy compared with manual classification

a CT: computed tomography.

b AUC: area under the curve.

c FDG PET: Fluorodeoxyglucose positron emission tomography.

d TNM: tumor, nodes, metastasis.

e K-M: Kaplan Meier.

### Quality Appraisal

**Figure 2.**
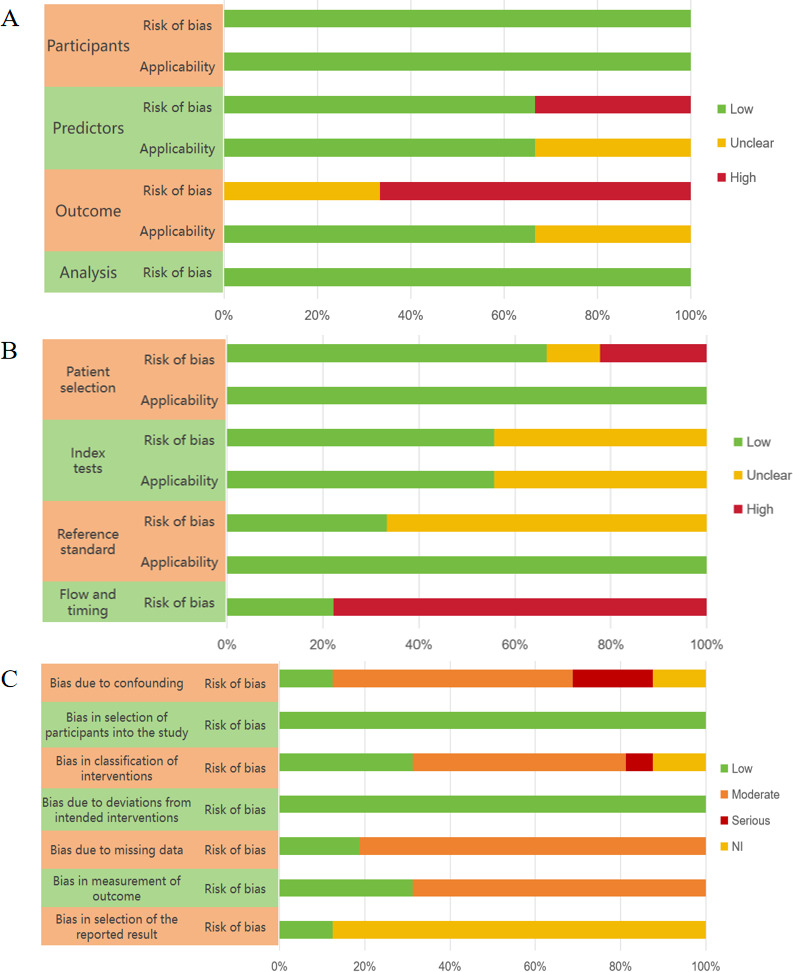
(A) The quality appraisal for 3 predictive studies with PROBAST (Prediction model Risk Of Bias Assessment Tool). (B) The quality appraisal for 9 diagnostic studies with QUADAS-2 (Quality Assessment of Diagnostic Accuracy Studies-2). (C) The quality appraisal for 16 intervention trials with ROBINS-I (Risk Of Bias In Non-randomized Studies - of Interventions).

The included studies were categorized based on their research objectives, and quality was assessed using corresponding appraisal tools (Multimedia Appendix 1). The kappa values of the 2 researchers were 0.84. Furthermore, 3 predictive modeling studies [35,43,44] were evaluated using the PROBAST tool (Figure 2A). These studies showed low risk of bias regarding data sources, populations, and methodologies but exhibited a potentially high risk in predictor and outcome domains. In total, 10 diagnostic studies [33,34,36-39,41,42,47] were assessed using QUADAS-2 (Figure 2B). While most demonstrated good applicability, the overall risk of bias remained unclear. Furthermore, 16 intervention studies [20-32,40,45,46] were appraised using the ROBINS-I tool (Figure 2C), showing low risk of bias in participant selection and intervention assignment, but unclear or high risk in other domains. Among them, 29% (18/63) of conference papers and preprints have a high-risk or unclear bias risk, while 26% (34/133) of journal papers have a high risk or unclear bias risk.

### Other Aspects

In addition, we examined whether the included studies reported human oversight, addressed safety considerations, and acknowledged limitations. In total, 26 (93%) studies [20-22,24,26-47] reported human involvement in system design, operation, or evaluation. Only 6 (21%) studies [20,31,33,38,42,43] explicitly addressed issues related to information security or data privacy. Furthermore, 20 (71%) studies [20-26,31-33,35,37-43,46,47] clearly stated their limitations.

## Discussion

### Principal Findings

**Figure 3.**
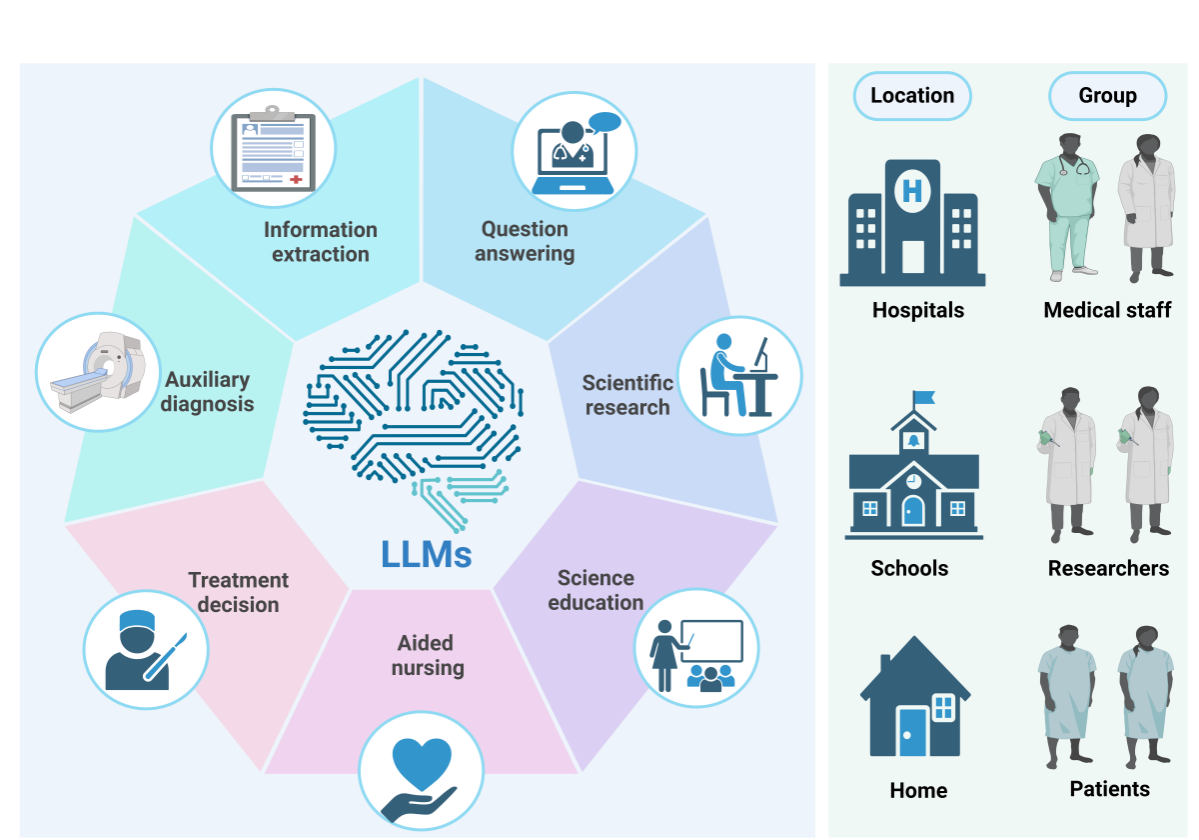
Applications of large language models in lung cancer. LLM: large language model.

Through a systematic review of 28 studies [20-47], we identified 7 primary application domains of LLMs in LC: auxiliary diagnosis, information extraction, question answering, scientific research, medical education, nursing support, and treatment decision-making (Figure 3). These domains often overlap in real-world practice—for instance, information extraction frequently supports diagnostic processes, while question-answering is commonly applied in science communication and patient education (Table 2).

### Applications of LLMs in LC

LLMs can extract clinical features by applying natural language processing methods. Therefore, many studies have used LLMs to extract and analyze information from electronic medical records [48], CT reports [21,22], and pathological reports [33,36] related to LC. This not only enables the diagnosis of clinical staging, histological type, lung-RADS (Reporting and Data System) score, and metastasis sites of LC, but also leverages their reasoning ability for diagnosis and prediction, such as lymph node metastasis [35] and malignancy degree of lung nodules [49]. This highlights the potential of LLMs in LC diagnosis, especially for early screening. Early diagnosis of LC can effectively improve survival rates [50], and mass LC screening achieves a high detection rate of early-stage LC [51], but it is time-consuming and labor-intensive. Ding et al [52] applied ChatGPT to automatically generate medical records during lung nodule screening sessions and integrated it into a WeChat (Tencent Holdings Limited) applet to streamline the consultation process. Singh et al [53] applied ChatGPT and Gemini (Google AI) to generate lung-RADS scores based on low-dose CT reports for LC screening, achieving up to 83.6% accuracy. A systematic review of LLMs in gastroenterology [54] similarly demonstrated the potential applications of LLMs in gastrointestinal endoscopy and the screening of precancerous lesions. Although LLMs still face challenges, such as insufficient extraction performance for complex tasks and hallucinations [55], the results of the study by Jong et al [42] also indicate that using LLMs in place of medical professionals for LC staging is not currently supported. However, with ongoing updates to training data and continuous upgrading and optimization of LLMs, we remain optimistic about their future performance in assisting with LC diagnosis and early screening.

Given the interactive nature and vast data reserves of LLMs, many studies have evaluated their application in knowledge question answering [27-32]. They have been widely applied in disseminating general knowledge about LC. With the refinement and diversification of training data and the development of multimodal large models, LLMs have shown improved capabilities in processing visual information [56]. Under carefully designed prompts and instructions, several studies have found that LLMs can perform preliminary analyses of medical images and textual data and, within controlled research settings, offer diagnostic and therapeutic suggestions for LC. Examples include providing initial recommendations for subsequent treatment options in newly diagnosed or suspected patients with NSCLC [57], generating more detailed chemotherapy [58] or radiotherapy [59] plans, and predicting outcome indicators such as overall survival [60] and radiotherapy-induced stress responses [44], thereby assisting treatment and nursing decision-making in research contexts [45]. Furthermore, LLMs pretrained on multilingual corpora have demonstrated potential in transcribing or translating LC radiology reports [61] and surgical records [62] to support multicenter clinical research. It should be noted, however, that most existing evidence is derived from retrospective analyses or small-sample, single-center studies. Robust prospective, multicenter clinical validation remains lacking, and systematic assessments of model interpretability, bias, and safety are still insufficient. Therefore, the reliability and generalizability of these methods in routine clinical practice require further confirmation.

The natural language processing and named entity recognition capabilities of LLMs can not only benefit clinicians and patients in clinical practice but also improve researchers’ efficiency. Devi et al [47] used GPT-3.5-turbo to classify patients with NSCLC based on pathological reports to determine their eligibility for clinical trials, assisting researchers with eligibility screening. Kyeryoung et al [25] used GPT-4 to extract safety and efficacy information from clinical trial abstracts and convert it into computable data for comparative analysis across large clinical trial datasets. Liu et al [63] used LLaMA 3.1 (Meta AI) to generate clinical trial annotations, enabling oncologists to stay fully updated with the latest oncology data presented at medical conferences and in journal publications. Similarly, Yuan et al [64] constructed and evaluated 3 machine learning models for predicting LC survival using an LLM-based advanced data analysis approach, making advanced analytics accessible to nontechnical health care professionals.

From the above, it is evident that the current applications of LLMs in LC span multiple stages of care, from early screening and diagnosis to treatment planning, patient follow-up, and research support. However, their maturity, evidence base, and clinical readiness vary substantially. Diagnostic and screening tools are the most developed, yet most rely on retrospective datasets and single-center studies, with limited prospective, multicenter clinical validation. Similarly, treatment planning applications show promise in integrating patient-specific data with clinical guidelines, but they also lack large-scale, prospective evaluations to confirm safety, effectiveness, and adaptability to evolving oncology standards. Patient follow-up and supportive care applications are even less developed, despite their potential to improve adherence, symptom management, and long-term quality of life. These stages are often complex due to diverse patient needs, variable follow-up schedules, and sensitive data management requirements, which may explain their slower technological adoption. Research-support tools, such as automated trial eligibility screening or survival prediction, demonstrate potential for improving efficiency, but their accuracy and reproducibility in real-world practice remain uncertain.

Based on these observations, we identify 3 research priorities. First, rigorous prospective, multicenter clinical validation of both diagnostic or screening and treatment planning applications to ensure generalizability and safety. Second, targeted development of patient follow-up and supportive care applications to address gaps in long-term management and patient engagement. Third, improvement of model interpretability, bias mitigation, and integration strategies to enable safe deployment across diverse health care systems. Addressing these gaps will be essential for the effective integration of LLMs into full-cycle LC management.

### Limitations of LLMs and Future Directions in LC

Clinical decision-making for LC in practice is driven by multimodal data, including clinical notes, radiological images, and pathological features. This implies that artificial intelligence tools capable of effectively integrating multimodal data hold significant potential for advancing clinical treatment of LC [65]. However, the research reviewed in this article still primarily focuses on text processing. Although efforts have been made to explore other data modalities, including CT images [34], pathological images [39], and bioinformatics data [46], the accuracy of their outputs has yet to match that of text-based outputs. Furthermore, studies indicate that deep learning models specialized in image processing, such as Convolutional Neural Networks, outperform LLMs in classifying LC cytology images [66]. Therefore, researchers tend to combine LLMs with other deep learning models for multimodal data analysis [38,43]. Nevertheless, the development and advancement of multimodal LLMs remain a key trend. Currently, OpenAI has taken the lead by launching ChatGPT-4o and ChatGPT-4V, spearheading the application boom of multimodal LLMs. In the future, LLMs are expected to overcome single-modality limitations on a large scale and enhance accurate diagnosis and treatment of LC by integrating multimodal reasoning capabilities across medical images, genomic data, biological molecular information, and even audio and video.

Existing research on LC predominantly uses general LLMs, such as ChatGPT and LLAMA-2, which are trained on public databases and experience slow knowledge base updates. These models may have gaps in domain-specific LC knowledge, and their outputs are prone to hallucinations and insufficient citations [67]. In recent years, many large models targeting specific tasks within clinical specialties have also emerged. For example, Med-PaLM 2 [68], which excels at lengthy medical question-answering; BioBERT [69], which specializes in biomedical texts; and ClinicalBERT [70], which focuses on clinical texts. However, studies have found that their performance on cardiac surgery knowledge quizzes [71] and precision LC treatment plans [72] is inferior to that of general LLMs with larger training parameter counts. Retrieval-augmented generation (RAG), a cutting-edge technology in large models, can reference reliable external knowledge (REK) to generate answers or content, enable real-time knowledge updates, and offer strong interpretability and customization capabilities [73]. Combining this technology with general large-scale models has resulted in more satisfactory outcomes. Built on Google’s Gemini Pro LLM, MEREDITH uses RAG and chain-of-thought reasoning. MEREDITH was enhanced to incorporate clinical studies on drug response within specific tumor types, trial databases, drug approval status, and oncologic guidelines. The precise treatment recommendations it provides for tumors closely align with expert advice [74]. Tozuka et al [75] summarized the current LC staging guidelines in Japan and supplied these as REK to NotebookLM, a RAG-equipped LLM. NotebookLM achieved 86% diagnostic accuracy in LC staging experiments, outperforming GPT-4o, which recorded 39% accuracy with REK and 25% without. In addition, appropriate prompt engineering can enhance the performance of general-purpose LLMs on specific tasks. Most of the studies included in our review used directives, prompt templates, and fine-tuning. Prompt templates often incorporated role descriptions, case examples, task requirements, LC-specific knowledge, and formatting instructions. Fine-tuning involves retraining a pretrained LLM (eg, ChatGPT and BERT) using labeled data for a specific task or domain to improve its performance on domain-specific tasks [76]. A study by Arzideh et al [77], comparing the extraction of clinical entities from unstructured medical records of patients with LC, found that a fine-tuned BERT model using annotated data achieved a higher *F*_1_-score than an instruction-based LLM. Similarly, Zhu et al [78] developed an open-source, oncology-specific LLM using a stacked alignment and fine-tuning process, which outperformed ChatGPT on medical benchmarks and achieved an area under the receiver operating characteristic curve of 0.95 for LC detection.

The studies included in this paper all used open-source LLMs; however, when deploying open-source LLMs in the cloud, issues related to data security and privacy protection are inevitable. Only 6 studies[20,31,33,38,42,43] have explicitly proposed specific data security measures, including legal constraints, such as the Health Insurance Portability and Accountability Act (HIPAA) [20] or standard protocols [38], data access restrictions [33], and data anonymization [41,43]. With the widespread adoption of LLMs in medical settings and growing awareness of data security, hospitals with significant application demands opt to deploy open-source LLMs locally, enabling models and data to operate entirely within the hospital intranet and thereby avoiding risks associated with cloud transmission [79]. They also mitigate data leakage risks through methods such as data anonymization and deidentification [80], federated learning [81,82], and differential privacy [83], among others. In the future, continued technological advancements and regulatory improvements, strengthened data supervision mechanisms, and a balanced approach between cost and performance will be essential to protect patient privacy.

At the same time, it should be acknowledged that LLMs cannot fully replace medical professionals, and it is necessary to clarify the responsibility attribution of LLMs in real clinical scenarios. Ethical frameworks should be established based on the needs of different medical scenarios and acceptable thresholds for patients and applied in a targeted manner [84]. Key applications with low risk of harm to patients’ health can be prioritized, such as patient registration codes [85], screening [37,86], and extraction of key information from medical records [87]. Through a “human-on-the-loop” human-machine collaboration model, reinforcement learning techniques are introduced to optimize prompt strategies and model decisions, enhance model transparency and clinician engagement, and strengthen human oversight.

### Limitations of This Systematic Review

This review includes studies published up to January 1, 2025. Due to the rapid development of LLMs and fast publication cycles, some recent findings may have been missed. To address this, we expedited manuscript preparation and included several additional studies from the past 7 months (from January to July 2025) in the discussion. To ensure the comprehensiveness and relevance of this review, we included all studies that provided complete data and full-text availability. However, some of the included conference papers and preprints may not have undergone peer review, potentially affecting the reliability of the findings. Nonetheless, our quality assessment indicated that their risk of bias did not differ significantly from that of peer-reviewed journal articles. Although 6 databases were searched, relevant studies outside these sources may have been overlooked. We also limited inclusion to English-language articles, which may affect generalizability, although only 2 non-English articles were excluded. Meanwhile, research conducted across different countries may be affected by population diversity and bias in training datasets. Unlike traditional reviews of clinical interventions, this study applied different quality assessment methods tailored to various application scenarios. Some criteria relied on subjective judgment, and the complexity of the process may have introduced bias. To minimize this, 2 researchers (SC and ZL) assessed studies independently, discrepancies were resolved with a third reviewer, and final decisions were validated by 2 experts (LG and KH).

### Conclusions

In summary, this systematic review offers an overview of the applications and research involving LLMs in LC, accompanied by a quality assessment. LLMs can assist physicians in interpreting test reports, delivering diagnostic and treatment recommendations, and supporting education, research, and public outreach efforts. The development of multimodal models, data quality, privacy-preserving mechanisms, and advanced LLM architectures is key to integrating these technologies into the full-cycle management of LC care. Within an ethical framework and under appropriate human oversight, future efforts should focus on validating LLM applications in real-world clinical settings and the inclusion of underrepresented populations to ensure population diversity, ultimately promoting their development toward greater specialization, accuracy, and patient-centeredness.

## Supplementary material

10.2196/74177 Multimedia Appendix 1 Specific quality evaluation items and results.

10.2196/74177 Checklist 1 PRISMA checklist.

## Abbreviations:

CT: computed tomography
GI: gastrointestinal
HIPAA: Health Insurance Portability and Accountability Act
LC: lung cancer
LLM: large language model
NSCLC: non-small cell carcinoma
OS: overall survival
PRISMA: Preferred Reporting Items for Systematic Reviews and Meta-Analyses
PROBAST: Prediction model Risk Of Bias Assessment Tool
PROSPERO: Prospective Register of Systematic Reviews
QUADAS-2: Quality Assessment of Diagnostic Accuracy Studies-2
RADS: Reporting and Data System
RAG: retrieval-augmented generation
REK: reliable external knowledge
ROBINS-I: Risk Of Bias In Non-randomized Studies - of Interventions
