# Supplementary material for: Large Language Models in Lung Cancer: Systematic Review
Source: J Med Internet Res. 2025 Sep 30;27:e74177. doi: 10.2196/74177 (PMC12483341; doi:10.2196/74177)
Supplement: Multimedia Appendix 1 [file jmir-v27-e74177-s001.docx]

**Multimedia Appendix 2**

Risk of bias

Table S1: The results of the risk of bias assessment according to the PROBAST tool.

| **DOMAIN 1 Participants** | | | |
| --- | --- | --- | --- |
|  | Dong 2024 | Hu 2024b | Sangwoon 2024 |
| 1.1 Were appropriate data sources used? | Y | Y | Y |
| 1.2 Were all inclusions and exclusions of participants appropriate? | Y | Y | Y |
| **Risk of bias introduced by selection of participants** | Low | Low | Low |
| **Concern that the included participants and setting do not match the review question** | Low | Low | Low |
| **DOMAIN 2: Predictors** | | | |
|  | Dong 2024 | Hu 2024b | Sangwoon 2024 |
| 2.1 Were predictors defined and assessed in a similar way for all participants? | Y | Y | Y |
| 2.2 Were predictor assessments made without knowledge of outcome data? | Y | Y | Y |
| 2.3 Are all predictors available at the time the model is intended to be used? | PN | Y | Y |
| **Risk of bias introduced by predictors or their assessment** | High | Low | Low |
| **Concern that the definition, assessment or timing of predictors in the model do not match the review question** | Unclear | Low | Low |
| **DOMAIN 3: Outcome** | | | |
|  | Dong 2024 | Hu 2024b | Sangwoon 2024 |
| 3.1 Was the outcome determined appropriately? | Y | Y | Y |
| 3.2 Was a pre-specified or standard outcome definition used? | Y | Y | PY |
| 3.3 Were predictors excluded from the outcome definition? | PN | PN | PY |
| 3.4 Was the outcome defined and determined in a similar way for all participants? | Y | Y | Y |
| 3.5 Was the outcome determined without knowledge of predictor information? | PN | PN | PY |
| 3.6 Was the time interval between predictor assessment and outcome determination  appropriate? | Y | Y | PY |
| **Risk of bias introduced by the outcome or its determination** | High | High | Unclear |
| **Concern that the outcome, its definition, timing or**  **determination do not match the review question** | Low | Low | Unclear |
| **DOMAIN 4: Analysis** | | | |
|  | Dong 2024 | Hu 2024b | Sangwoon 2024 |
| 4.1 Were there a reasonable number of participants with the outcome? | Y | Y | PY |
| 4.2 Were continuous and categorical predictors handled appropriately? | Y | Y | PY |
| 4.3 Were all enrolled participants included in the analysis? | Y | Y | Y |
| 4.4 Were participants with missing data handled appropriately? | Y | PY | PY |
| 4.5 Were complexities in the data (e.g. censoring, competing risks, sampling of controls) accounted for appropriately? | Y | PY | Y |
| 4.6 Were relevant model performance measures evaluated appropriately? | Y | Y | Y |
| 4.7 Were model overfitting and optimism in model performance accounted for? | Y | Y | Y |
| **Risk of bias introduced by the analysis** | Low | Low | Low |

Tips:Signaling questions are rated as yes (Y), probably yes (PY), probably no (PN), no (N) or no information (NI). All signaling questions are phrased so that “yes” indicates absence of bias. Any signaling question rated as “no” or “probably no” flags the potential for bias.

Table S2: The results of the risk of bias assessment according to the QADAS-2 tool.

| **DOMAIN 1: Patient selection** | | | | | | | | | | |
| --- | --- | --- | --- | --- | --- | --- | --- | --- | --- | --- |
|  | | Hyeongmin 2024 | Reza 2024 | Huang 2024 | Koichiro 2024 | Gopi 2024 | Sneha 2024 | Jong 2024 | Arti 2024 | Qu 2024 |
| 1.1 Was a consecutive or random sample of patients enrolled? | | Yes | Yes | No | Yes | No | Yes | Yes | No | Unclear |
| 1.2 Did the study avoid inappropriate exclusions? | | Yes | Yes | Yes | Yes | Yes | Yes | Yes | Yes | Yes |
| **Could the selection of patients have introduced bias?** | | Low | Low | High | Low | Low | Low | Low | High | Unclear |
| **Is there concern that the included patients do not match the review question?** | | Low | Low | Low | Low | Low | Low | Low | Low | Low |
| **DOMAIN 2: Index test(s)** | | | | | | | | | | |
|  | | Hyeongmin 2024 | Reza 2024 | Huang 2024 | Koichiro 2024 | Gopi 2024 | Sneha 2024 | Jong 2024 | Arti 2024 | Qu 2024 |
| 2.1 If a threshold was used, was it pre-specified? | | Yes | Yes | Yes | Yes | Yes | Unclear | Unclear | Unclear | Unclear |
| **Could the conduct or interpretation of the index test**  **have introduced bias?** | | Low | Low | Low | Low | Low | Unclear | Unclear | Unclear | Unclear |
| **Is there concern that the index test, its conduct, or**  **interpretation differ from the review question?** | | Low | Low | Low | Low | Low | Unclear | Unclear | Unclear | Unclear |
| **DOMAIN 3: Reference standard** | | | | | | | | | | |
|  |  | Hyeongmin 2024 | Reza 2024 | Huang 2024 | Koichiro 2024 | Gopi 2024 | Sneha 2024 | Jong 2024 | Arti 2024 | Qu 2024 |
| 3.1 Is the reference standard likely to correctly classify the target  condition? | | Yes | Yes | Yes | Yes | Yes | Yes | Yes | Yes | Yes |
| 3.2 Were the reference standard results interpreted without  knowledge of the results of the index test? | | Unclear | Unclear | Unclear | Yes | Yes | Unclear | Yes | Unclear | Unclear |
| **Could the reference standard, its conduct, or its**  **interpretation have introduced bias?** | | Unclear | Unclear | Unclear | Low | Low | Unclear | Low | Unclear | Unclear |
| **Is there concern that the target condition as defined by the reference standard does not match the review**  **question?** | | Low | Low | Low | Low | Low | Low | Low | Low | LOW |
| **DOMAIN 4: Flow and timing** | | | | | | | | | | |
|  | | Hyeongmin 2024 | Reza 2024 | Huang 2024 | Koichiro 2024 | Gopi 2024 | Sneha 2024 | Jong 2024 | Arti 2024 | Qu 2024 |
| 4.1 Did all patients receive a reference standard and the same reference standard? | | Yes | Yes | Yes | Yes | Yes | Yes | Yes | Yes | Yes |
| 4.1 Were all patients included in the analysis? | | No | No | No | No | No | No | No | Yes | Yes |
| **Could the patient flow have introduced bias?** | | High | High | High | High | High | High | High | Low | Low |

Tips:Signaling questions are rated as Yes, No or Unclear.If the answers to all signaling questions are "yes", the risk of bias is low; If the answer to a signal question is "no", there is a risk of bias; Other cases were judged as "unclear".

Table S3: The results of the risk of bias assessment according to the ROBINS-I tool.

| **DOMAIN 1: Bias due to confounding** | | | | | | | | | | | | | | | | |
| --- | --- | --- | --- | --- | --- | --- | --- | --- | --- | --- | --- | --- | --- | --- | --- | --- |
|  | **Kriti 2024** | **Fabiana 2024** | **Ferrari 2024** | **Matthias 2023** | **Adem 2024** | **Hana 2023** | **Hu 2024a** | **James 2024** | **Wang 2024** | **Julian 2023** | **Amir 2023** | **Niu 2024** | **Narmada 2023** | **Lyu 2023** | **Kyeryoung 2024** | **Dimitrios 2023** |
| 1.1 Is there potential for confounding of the effect of intervention in this study? | PY | PN | N | PY | PY | PY | PN | PY | PY | PY | PY | PY | Y | Y | Y | Y |
| 1.2 Was the analysis based on splitting participants' follow up time according to intervention received? | N | N | N | N | N | N | N | N | N | N | N | N | N | N | N | N |
| 1.3. Did the authors use an appropriate analysis method that controlled for all the important confounding domains? | Y | PY | PN | PY | PY | PY | Y | PY | PN | PY | Y | Y | PY | PN | PY | PY |
| 1.4. If Y/PY to 1.3: Were confounding domains that were controlled for measured validly and reliably by the variables available in this study? | Y | PY |  | PY | PY | PY | Y | PY |  | PY | PY | PY | PY |  | PY | PY |
| 1.5. Did the authors control for any post-intervention variables that could have been affected by the intervention? | PN | PN | PN | PN | PN | PN | PN | PN | PN | PN | NI | NI | N | PN | N | N |
| 1.6. Did the authors use an appropriate analysis method that controlled for all the important confounding domains and for time-varying confounding? | PN | PN | PN | PN | PN | PN | PN | PN | PN | PN | NI | NI | N | PN | N | N |
| **Risk of bias judgement** | Low | Moderate | Serious | Moderate | Moderate | Moderate | Low | Moderate | Serious | Moderate | NI | NI | Moderate | Serious | Moderate | Moderate |
| **DOMAIN 2: Bias in selection of participants into the study** | | | | | | | | | | | | | | | | |
|  | **Kriti 2024** | **Fabiana 2024** | **Ferrari 2024** | **Matthias 2023** | **Adem 2024** | **Hana 2023** | **Hu 2024a** | **James 2024** | **Wang 2024** | **Julian 2023** | **Amir 2023** | **Niu 2024** | **Narmada 2023** | **Lyu 2023** | **Kyeryoung 2024** | **Dimitrios 2023** |
| 2.1. Was selection of participants into the study (or into the analysis) based on participant characteristics observed after the start of intervention? | N | N | N | N | N | N | N | N | N | N | N | N | N | N | N | N |
| 2.4. Do start of follow-up and start of intervention coincide for most participants? | Y | Y | NI | NI | Y | Y | NI | Y | NI | Y | Y | Y | PY | NI | NI | NI |
| **Risk of bias judgement** | Low | Low | Low | Low | Low | Low | Low | Low | Low | Low | Low | Low | Low | Low | Low | Low |
| **DOMAIN 3: Bias in classification of interventions** | | | | | | | | | | | | | | | | |
|  | **Kriti 2024** | **Fabiana 2024** | **Ferrari 2024** | **Matthias 2023** | **Adem 2024** | **Hana 2023** | **Hu 2024a** | **James 2024** | **Wang 2024** | **Julian 2023** | **Amir 2023** | **Niu 2024** | **Narmada 2023** | **Lyu 2023** | **Kyeryoung 2024** | **Dimitrios 2023** |
| 3.1 Were intervention groups clearly defined? | Y | N | N | N | N | N | NI | N | NI | N | PY | PY | PN | PN | PY | Y |
| 3.2 Was the information used to define intervention groups recorded at the start of the intervention? | Y | Y | Y | Y | Y | Y | NI | Y | NI | Y | Y | Y | PN | PY | Y | Y |
| 3.3 Could classification of intervention status have been affected by knowledge of the outcome or risk of the outcome? | N | N | N | N | N | N | NI | N | NI | N | PY | PY | PN | PN | PN | N |
| **Risk of bias judgement** | Low | Moderate | Moderate | Moderate | Moderate | Moderate | NI | Moderate | NI | Moderate | Low | Low | Serious | Moderate | Low | Low |
| **DOMAIN 4: Bias due to deviations from intended interventions** | | | | | | | | | | | | | | | | |
|  | **Kriti 2024** | **Fabiana 2024** | **Ferrari 2024** | **Matthias 2023** | **Adem 2024** | **Hana 2023** | **Hu 2024a** | **James 2024** | **Wang 2024** | **Julian 2023** | **Amir 2023** | **Niu 2024** | **Narmada 2023** | **Lyu 2023** | **Kyeryoung 2024** | **Dimitrios 2023** |
| 4.1. Were there deviations from the intended intervention beyond what would be expected in usual practice? | PN | PN | PN | PN | PN | PN | PN | PN | PN | PN | PN | PN | PN | PN | PN | PN |
| **Risk of bias judgement** | Low | Low | Low | Low | Low | Low | Low | Low | Low | Low | Low | Low | Low | Low | Low | Low |
| **DOMAIN 5: Bias due to missing data** | | | | | | | | | | | | | | | | |
|  | **Kriti 2024** | **Fabiana 2024** | **Ferrari 2024** | **Matthias 2023** | **Adem 2024** | **Hana 2023** | **Hu 2024a** | **James 2024** | **Wang 2024** | **Julian 2023** | **Amir 2023** | **Niu 2024** | **Narmada 2023** | **Lyu 2023** | **Kyeryoung 2024** | **Dimitrios 2023** |
| 5.1 Were outcome data available for all, or nearly all, participants? | PN | PN | PN | PN | N | N | N | N | N | N | N | N | N | N | N | N |
| 5.2 Were participants excluded due to missing data on intervention status? | PY | PY | PY | PY | PY | PY | Y | PY | Y | PY | PY | PY | PY | PY | Y | PY |
| 5.3 Were participants excluded due to missing data on other variables needed for the analysis? | PN | PN | PN | PN | PN | PN | PN | PN | N | PN | PN | PN | PN | PN | N | PN |
| 5.4 If PN/N to 5.1, or Y/PY to 5.2 or 5.3: Are the proportion of participants and reasons for missing data similar across interventions? | NI | NI | NI | NI | NI | NI | NI | NI | NI | NI | NI | NI | NI | NI | NI | NI |
| 5.5 If PN/N to 5.1, or Y/PY to 5.2 or 5.3: Is there evidence that results were robust to the presence of missing data? | NI | NI | NI | NI | NI | NI | NI | NI | NI | NI | NI | NI | NI | NI | NI | NI |
| **Risk of bias judgement** | Moderate | Moderate | Moderate | Moderate | Moderate | Moderate | Low | Moderate | Low | Moderate | Moderate | Moderate | Moderate | Moderate | Low | Moderate |
| **DOMAIN 6: Bias in measurement of outcome** | | | | | | | | | | | | | | | | |
|  | **Kriti 2024** | **Fabiana 2024** | **Ferrari 2024** | **Matthias 2023** | **Adem 2024** | **Hana 2023** | **Hu 2024a** | **James 2024** | **Wang 2024** | **Julian 2023** | **Amir 2023** | **Niu 2024** | **Narmada 2023** | **Lyu 2023** | **Kyeryoung 2024** | **Dimitrios 2023** |
| 6.1 Could the outcome measure have been influenced by knowledge of the intervention received? | N | Y | Y | N | N | N | Y | N | Y | N | N | N | N | Y | Y | PY |
| 6.2 Were outcome assessors aware of the intervention received by study participants? | N | PY | Y | PY | Y | Y | PY | Y | Y | Y | Y | Y | Y | Y | PY | PY |
| 6.3 Were the methods of outcome assessment comparable across intervention groups? | Y | Y | Y | Y | PY | PY | PY | PY | N | PY | PY | PY | PY | N | PY | N |
| 6.4 Were any systematic errors in measurement of the outcome related to intervention received? | N | N | PY | PY | PY | PY | PY | PY | Y | PY | PY | PY | PY | Y | PY | Y |
| **Risk of bias judgement** | Low | Low | Low | Moderate | Moderate | Moderate | Low | Moderate | Moderate | Moderate | Moderate | Moderate | Moderate | Moderate | Low | Moderate |
| **DOMAIN 7: Bias in selection of the reported result** | | | | | | | | | | | | | | | | |
|  | **Kriti 2024** | **Fabiana 2024** | **Ferrari 2024** | **Matthias 2023** | **Adem 2024** | **Hana 2023** | **Hu 2024a** | **James 2024** | **Wang 2024** | **Julian 2023** | **Amir 2023** | **Niu 2024** | **Narmada 2023** | **Lyu 2023** | **Kyeryoung 2024** | **Dimitrios 2023** |
| 7.1.multiple outcome measurements within the outcome domain? | N | N | NI | NI | NI | NI | NI | NI | NI | NI | NI | NI | NI | NI | NI | NI |
| 7.2 multiple analyses of the intervention-outcome relationship? | PY | PN | NI | NI | NI | NI | NI | NI | NI | NI | NI | NI | NI | NI | NI | NI |
| 7.3 different subgroups? | N | N | NI | NI | NI | NI | NI | NI | NI | NI | NI | NI | NI | NI | NI | NI |
| **Risk of bias judgement** | Low | Low | NI | NI | NI | NI | NI | NI | NI | NI | NI | NI | NI | NI | NI | NI |

Tips:The answer options for each item were Yes (Y), Probably yes (PY), Probably no (PN), No (N), and No information (NI). After answering and evaluating each item, the risk of bias in the corresponding field should be assessed as “low, moderate, serious, critical, or no information” according to the criteria established in advance.
